# Leptomeningeal metastases from a primary central nervous system melanoma: a case report and literature review

**DOI:** 10.1186/1477-7819-12-265

**Published:** 2014-08-20

**Authors:** Zhenyu Pan, Guozi Yang, Yongxiang Wang, Tingting Yuan, Yan Gao, Lihua Dong

**Affiliations:** Department of Radiotherapy, Norman Bethune First Hospital, Jilin University, 71 Xinmin Street, Changchun, 130021 China; Department of Clinical Laboratory, Norman Bethune First Hospital, Jilin University, 71 Xinmin Street, Changchun, 130021 China; Department of Radiology, Norman Bethune First Hospital, Jilin University, 71 Xinmin Street, Changchun, 130021 China

**Keywords:** Central nervous system, Primary melanoma, Leptomeningeal metastases

## Abstract

Primary central nervous system (CNS) melanoma is a type of rare and aggressive tumor that can easily spread to the leptomeninges, and in fact, leptomeningeal metastasis is one of the most serious complications in patients with this carcinoma. Prognosis is extremely poor if a CNS melanoma has metastasized, and there are no effective treatments. Here, we present a case of a 37-year-old woman who presented with horizontal diplopia and progressive headache. Magnetic resonance imaging findings were consistent with the diagnosis of melanoma. The results of cytological examination of cerebrospinal fluid (CSF) showed malignant cells characteristic of melanoma. No extracranial lesions were observed. All of the available evidence confirmed a diagnosis of leptomeningeal metastases from a primary CNS melanoma. The patient received aggressive treatment, which consisted of concurrent radiotherapy and weekly intra-CSF methotrexate (MTX) followed by adjuvant monthly intra-CSF MTX. Her survival time was 13 months after diagnosis. This case report suggests that the modality of concurrent radiotherapy and weekly intra-CSF MTX followed by adjuvant monthly intra-CSF MTX may be used as the mainstay of treatment for such patients.

## Background

Primary central nervous system (CNS) melanoma is an uncommon disease and represents only 1% of all cases of melanoma [1] and 0.07% of all brain tumors [2]. Melanoblasts, the precursors of melanocytes, are of neural crest origin, and during development they migrate to the skin, uvea, mucous membranes and leptomeninges of the CNS. Primary CNS melanoma is thought to arise once melanocytes become neoplastic [3]. Because of its histogenetic characteristics, it frequently metastasizes to the leptomeninges, representing one of the most serious complications for cancer patients. Although a subset of patients with leptomeningeal metastases, particularly those with lymphoma or breast cancer, may survive for more than 12 months with a reasonable quality of life, leptomeningeal metastasis from solid tumors such as melanoma is associated with very poor prognosis. At present, intrathecal chemotherapy is standard treatment, but is not associated with a significant increase in survival, and at best is only palliative [4].

## Case presentation

**Figure 1:**
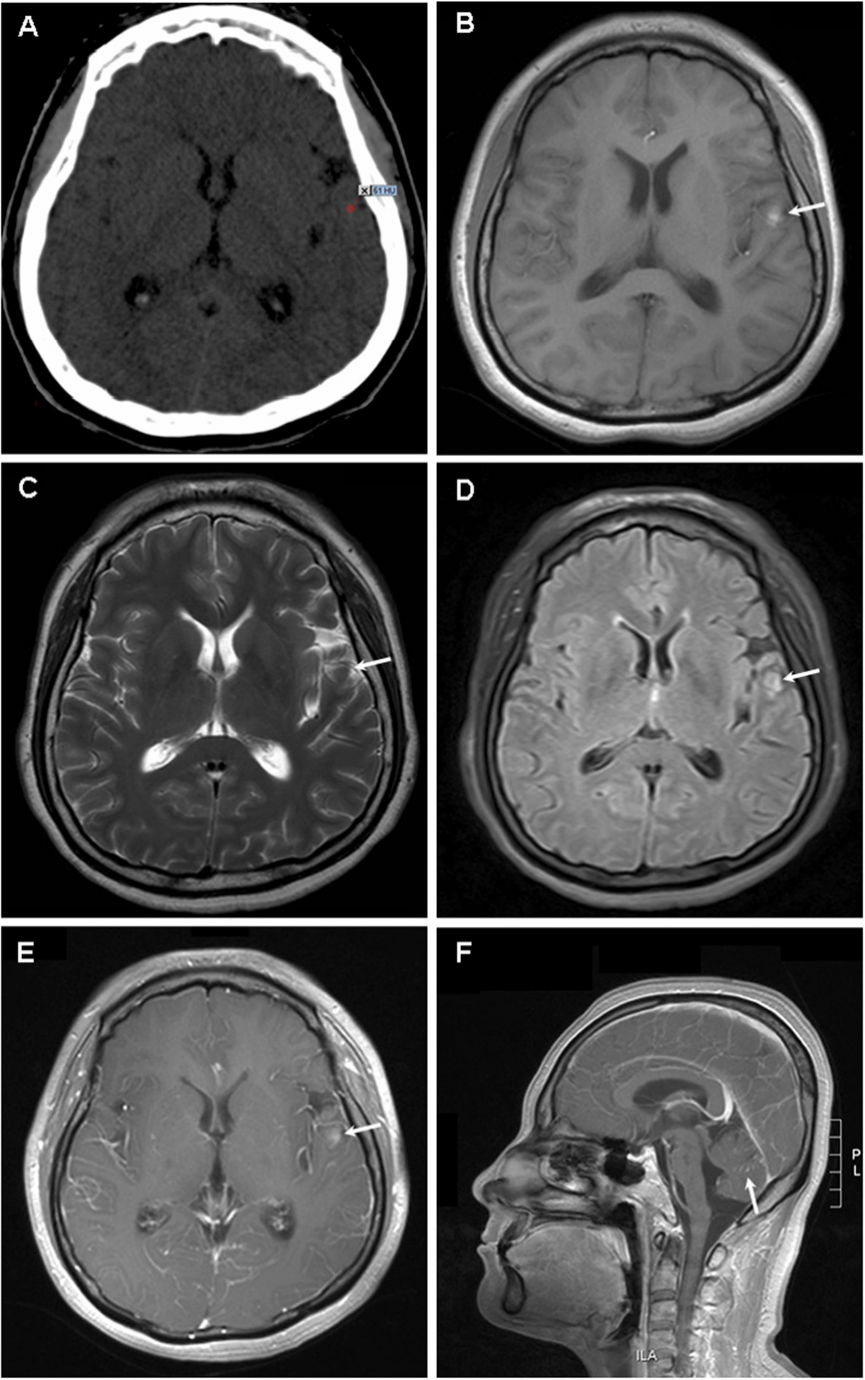
**Imaging findings for brain. (A)** Computed tomography image showing an isodense or slightly hyperdense lesion in the left temporal lobe (CT value: 45 to 61). Magnetic resonance image showing a 6-mm abnormal signal in the left temporal lobe, which was hyperintense on T1-weighted images **(B)**, hypointense on T2-weighted images **(C)**, slightly hyperintense on fluid-attenuated inversion recovery images **(D)**, and mild enhancement with gadolinium contrast **(E)**. Punctate and linear enhancement in the sulci and gyri of the cerebellum can be observed **(F)**.

A 37-year-old woman was referred to our hospital because of horizontal diplopia and progressive headache for 2 weeks. She had a medical history of non-frequent epilepsy as a child. Scattered melanocytic nevi with hair were observed on her skin when she was born, without darkened color, enlargement, pruritus or ulceration. Neurological examination on admission revealed right eye abduction. No other focal neurological signs, such as abnormal reflexes, sensory deficits or neck stiffness, were noted.Cranial computed tomography (CT) scans revealed an isodense or slight hyperdense lesion (CT value: 45 to 61) in the left temporal lobe (Figure 1A). Magnetic resonance imaging (MRI) of the brain demonstrated a 6-mm abnormal signal with paramagnetic qualities in the left temporal lobe, which shortened both T1 and T2 relaxation times. It was manifested as hyperintense on T1-weighted images (Figure 1B), hypointense on T2-weighted images (Figure 1C) and slightly hyperintense on fluid-attenuated inversion recovery images (Figure 1D), with mild enhancement with gadolinium contrast (Figure 1E). In addition, slightly hyperintense signals in the sulci and gyri of the bilateral frontotemporal lobe as well as punctate and linear enhancement in the sulci and gyri of the cerebellum were observed (Figure 1F). She also underwent chest and abdominal CT, which showed no abnormalities.

**Figure 2:**
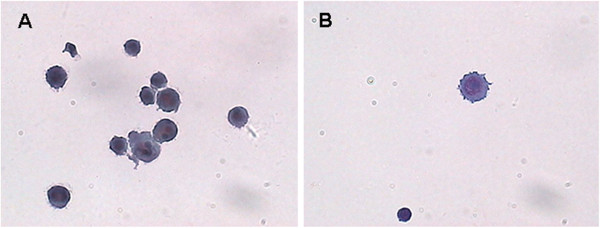
**Malignant melanoma cells in the cerebral spinal fluid. (A, B)** Round or oval tumor cells with different sizes were scattered, and presented as pseudopodia-like protrusions on cell membranes, with basophilic characteristics. Cells had round nuclei, large and distinct nucleoli, coarse and unevenly distributed chromatin, and particles in the nucleus or cytoplasm. (Liquid-based cytology, Papanicolaou staining, ×400).

A non-traumatic lumbar puncture was performed. The macroscopic appearance of the cerebrospinal fluid (CSF) was hematic and cloudy, and the opening pressure was 30.4 mmHg (5 to 15 mmHg). Biochemical analysis of the CSF revealed that the glucose level was 28.8 mg/dL (41.4 to 73.8) and the protein level was 66 mg/dL (15 to 45). The cells consisted of leucocytes (15 cells/mm^3^) and erythrocytes (110 cells/mm^3^). Malignant cells with the characteristics of melanoma were found by cytological examination of the CSF (Figure 2). No bacteria, viruses, parasites or other pathogenic microorganisms could be detected in the CSF.

The patient received aggressive treatment including concurrent whole brain radiotherapy (linear accelerator, 6 MV photon beam, 40 Gy in 20 fractions) and intra-CSF chemotherapy. Methotrexate (MTX) 15 mg and dexamethasone 5 mg were given intrathecally once weekly from the second day of radiotherapy, for a total of 8 weeks. On the eighth administration of intra-CSF MTX, CSF examination showed that the opening pressure was 18.2 mmHg, with a colorless transparent appearance. The glucose and protein levels were 28.8 mg/dL and 18.5 mg/dL, respectively. Cellular components consisted of leucocytes (20 cells/mm^3^) and erythrocytes (10 cells/mm^3^). No malignant cells were found in the CSF. Symptomatically, the patient was in complete remission. No severe treatment-related adverse events were noted, and her memory and cognitive ability had not obviously changed before and after treatment. Thereafter, it was suggested that she undergo monthly intra-CSF MTX. However, the patient herself refused the treatment on the fifth month. Headache and vomiting reappeared, but resolved following intra-CSF MTX (15 mg) monthly. Treatment was again interrupted until month 9, and recurrence was noted. She refused any examinations including brain MRI and CSF analysis. Unfortunately, re-administration of intra-CSF MTX was ineffective. The patient died from progression of the nervous system disease at 13 months after initial presentation with diplopia.

## Discussion

It seems reasonable that malignant melanoma can occur in any organ in which melanoblasts can normally be found. This includes the pia mater, skin and uvea. However, primary CNS melanoma is thought to be an uncommon pathology. It can be divided into two forms. The first invades the meninges diffusely, while the second causes nodular intraparenchymal lesions. There are frequent literature reports regarding melanoma of the nervous system, but in some cases it is not easy to differentiate the primary tumor from metastatic disease. Prognosis is extremely poor, especially in the presence of leptomeningeal metastasis. There are no effective treatments. To our knowledge, this is the first case of a patient receiving aggressive treatment that consisted of concurrent radiotherapy and weekly intra-CSF MTX followed by adjuvant monthly intra-CSF MTX. This treatment modality gave a relatively long survival time.

In contrast to brain metastases, which usually occur in the junction between gray and white matter, intraparenchymal nodular lesions of primary melanomas may occur in the brain at any leptomeningeal location [5]. The tumor in the present patient was located in the left temporal lobe adjacent to the Sylvian fissure close to the leptomeninges. This was consistent with the developing characteristics of primary CNS melanoma. The imaging features of the primary CNS melanoma were unique. Typically, a melanoma appears hyperdense on unenhanced CT, and after intravenous administration of iodinated contrast medium, uniform or ring enhancement is generally observed [1]. The MRI signal of a melanoma in the CNS depends on several factors. Studies of metastatic melanomas have demonstrated a trend toward shorter T1 and T2 relaxation times, with increasing melanin content ascribed to the paramagnetic effect of melanin [6], as demonstrated in the present case. These characteristics are not seen in other intracranial tumors, and can be used to confirm diagnosis [7]. In addition, spontaneous hemorrhage is frequently reported in melanoma because the tumor is rich in blood vessels, and can easily invade the vessels of the meninges, which also has a suggestive role in diagnosis [8].

CSF cytology may provide an important basis for diagnosis of leptomeningeal melanoma. The melanocytes present in the CSF appear uneven in size, with basophilia and an increased nucleocytoplasmic ratio. They are multinuclear and there are irregular pseudopodial apophyses on the cytomembrane. All of these are specific characteristics of melanoma cells.

Congenital melanocytic nevi are benign proliferations of cutaneous melanocytes and are usually defined as melanocytic lesions present at birth [9]. Individuals with congenital melanocytic nevi are at high risk of developing malignant melanoma [10, 11]. About 25% of patients with primary CNS melanoma have been reported to have congenital melanocytic nevi [12]. To render a diagnosis of primary CNS melanoma, there must be no evidence of the lesion elsewhere [13, 14]. The melanocytic nevi in the present patient were observed when she was born, but no signs of malignancy were found. In addition, no extracranial lesions were observed from the onset of disease to death. All of the available evidence confirmed a diagnosis of primary CNS melanoma.

Primary CNS melanoma is an aggressive type of tumor that may metastasize to other organs. Total resection or radiotherapy is the primary treatment for patients with local intraparenchymal lesions [1, 5], and may be associated with relatively long survival [15, 16]. However, the prognosis of metastatic leptomeningeal melanoma is very poor because of complete CNS involvement by tumor cells. Harstad *et al*. conducted a study with 110 patients with metastatic leptomeningeal melanoma and reported that the overall median survival was 10 weeks [17]. Intra-CSF chemotherapy, as a treatment modality for the CNS, can kill tumor cells in the CSF directly, and is the primary treatment for leptomeningeal neoplasms. MTX is the most effective drug used in intra-CSF chemotherapy for leptomeningeal metastases from solid tumors including melanoma [18]. The total CSF volume is 100 to 150 mL, and 10 to 15 mg of MTX once or twice weekly can be considered an effective cytotoxic concentration in the CSF [19]. The corticosteroids used in intra-CSF chemotherapy can effectively reduce the incidence of chemical meningitis and relieve nervous system toxicity induced by chemotherapeutic agents [19].

Involved-field radiotherapy at sites of symptomatic or bulky disease is an important treatment modality for the majority of patients with leptomeningeal neoplasm. Paolo *et al.* documented a patient with advanced melanoma and multiple metastases harboring the ^V600E^BRAF mutation, who underwent treatment with BRAF inhibitors [20]. The patient progressively deteriorated and died of leptomeningeal metastasis. However, another case presented by Lee *et al.* involved a patient with a similar disease who received treatment with BRAF inhibitors and whole brain radiotherapy (30 Gy in 10 fractions) [21]. Remission of symptoms was achieved and long-term survival was reported. These two distinct prognoses suggest that whole brain radiotherapy plays an important role in the treatment of leptomeningeal metastases. Radiotherapy in patients with cranial neuropathies is also indicated to reestablish normal CSF circulation to permit increased efficacy of intra-CSF chemotherapy, which has been shown to improve outcomes [18].

Chamberlain and Kormanik carried out a phase II study of combined modality therapy for leptomeningeal metastases due to melanoma, which enrolled 16 patients [22]. The majority of patients in that study underwent intraventricular chemotherapy after completion of radiotherapy. The overall survival of all patients was 2 to 8 months, with a median survival of 4 months; 75% of patients died as a result of progressive leptomeningeal metastases and/or systemic disease progression.

Based on previous studies, concurrent radiotherapy and intra-CSF chemotherapy were chosen for the present patient. Symptoms related to meningeal irritation were relieved quickly, and the patient survived for more than one year. No severe toxicity was observed. The therapeutic advantages of the present treatment modality are as follows: (1) MTX is an S-phase specific antineoplastic agent, and tumor cells in the G2 and M phases are more sensitive to radiotherapy than cells in other phases. Therefore, radiotherapy combined with intra-CSF MTX may improve the antitumor effects because of a synergistic effect on different phases of the cell cycle. (2) MTX has a sensitizing effect on radiotherapy [23]. (3) Whole brain radiotherapy may break the blood–brain barrier, which improves CSF circulation and is beneficial in distributing chemotherapeutic agents in the CSF [24].Unfortunately, the patient received sequential intra-CSF MTX at a local hospital after disease progression. Therefore, it is unclear if survival could have been further prolonged if the treatment had been changed or if systemic chemotherapy had been administered.

## Conclusions

In general, primary CNS melanoma is a rare disease associated with poor prognosis when the leptomeninges are involved. The treatment modality of concurrent radiotherapy and weekly intra-CSF MTX followed by adjuvant monthly intra-CSF MTX may be an efficacious combination. However, additional studies are needed to improve treatment and prognosis.

## Consent

Written informed consent was obtained from the next of kin of the patient for publication of this case report and any accompanying images. A copy of the written consent is available for review by the Editor-in-Chief of this journal.

## Abbreviations

CNS: Central nervous system
CSF: Cerebrospinal fluid
CT: Computed tomography
MRI: Magnetic resonance imaging
MTX: Methotrexate.

## References

[1] Greco Crasto S, Soffietti R, Bradac GB, Boldorini R (2001). Primitive cerebral melanoma: case report and review of the literature. SurgNeurol.

[2] Suzuki T, Yasumoto Y, Kumami K, Matsumura K, Kumami M, Mochizuki M, Suzuki H, Kojima H (2001). Primary pineal melanocytic tumor. Case report. J Neurosurg.

[3] Rahimi-Movaghar V, Karimi M (2003). Meningeal melanocytoma of the brain and oculodermalmelanocytosis (nevus of Ota): case report and literature review. SurgNeurol.

[4] Bruno MK, Raizer J (2005). Leptomeningeal metastases from solid tumours (meningeal carcinomatosis). Cancer Treat Res.

[5] Liubinas SV, Maartens N, Drummond KJ (2010). Primary melanocytic neoplasms of the central nervous system. J ClinNeurosci.

[6] Isiklar I, Leeds NE, Fuller GN, Kumar AJ (1995). Intracranial metastatic melanoma: correlation between MR imaging characteristics and melanin content. Am J Roentgenol.

[7] Somers KE, Almast J, Biemiller RA, Silberstein HJ, Johnson MD, Mohile NA (2013). Diagnosis of primary CNS melanoma with neuroimaging. J ClinOncol.

[8] Cliffor JR, Kirgis HD, Connolly ES (1975). Metastatic melanoma of the brain presenting as subarachnoid hemorrhage. South Med J.

[9] Grichnik JM, Rhodes AR, Sober AJ, Wolff K, Goldsmith LA, Katz SI, Gilchrest BA, Paller AS, Lefell DJ (2008). Benign neoplasias and hyperplasias of melanocytes. Fitzpatrick’s Dermatology in General Medicine. Volume 1.

[10] Zaal LH, Mooi WJ, Klip H, van der Horst CM (2005). Risk of malignant transformation of congenital melanocytic nevi: a retrospective nationwide study from The Netherlands. PlastReconstrSurg.

[11] Bittencourt FV, Marghoob AA, Kopf AW, Koenig KL, Bart RS (2000). Large congenital melanocytic nevi and the risk for development of malignant melanoma and neurocutaneousmelanocytosis. Pediatrics.

[12] Hoffman HJ, Freeman A (1967). Primary malignant leptomeningeal melanoma in association with giant hairy nevi. J Neurosurg.

[13] Hayward RD (1976). Malignant melanoma and cerebral nervous system. A guide for classification based on the clinical findings. J NeurolNeurosurg Psychiatry.

[14] Savitz MH (1987). Primary melanomas of the central nervous system. J Neurosurg.

[15] Nakagawa H, Hayakawa T, Niiyama K, Nii Y, Yoshimine T, Mori S (1989). Long-term survival after removal of primary intracranial malignant melanoma. Acta Neurochir (Wien).

[16] Rubino J, King WA, Quinn Marroquin CE, Verity A (1993). Primary pineal melanoma: case report. Neurosurgery.

[17] Harstad L, Hess KR, Groves MD (2008). Prognostic factors and outcomes in patients with leptomeningealmelanomatosis. NeuroOncol.

[18] Le Rhun E, Taillibert S, Chamberlain MC (2013). Carcinomatous meningitis: leptomeningeal metastases in solid tumors. SurgNeurolInt.

[19] Chamberain MC (2005). Neoplastic meningitis. J ClinOncol.

[20] Simeone E, De Maio E, Sandomenico F, Fulciniti F, Lastoria S, Aprea P, Staibano S, Montesarchio V, Palmieri G, Mozzillo N, Ascierto PA (2012). Neoplastic leptomeningitis presenting in a melanoma patient treated with dabrafenib (a V600EBRAF inhibitor): a case report. J Med Case Rep.

[21] Lee JM, Mehta UN, Dsouza LH, Guadagnolo BA, Sanders DL, Kim KB (2013). Long-term stabilization of leptomeningeal disease with whole-brain radiation therapy in a patient with metastatic melanoma treated with vemurafenib: a case report. Melanoma Res.

[22] Chamberlain M, Kormanik P (1996). Leptomeningeal metastases due to melanoma. Int J Oncol.

[23] Kim A, Lee JE, Jang WS, Lee SJ, Park S, Kang HJ, Lee SS (2012). A combination of methotrexate and irradiation promotes cell death in NK/T-cell lymphoma cells via down-regulation of NF-κB signaling. Leuk Res.

[24] Qin DX, Zheng R, Tang J, Li JX, Hu YH (1990). Influence of radiation on the blood–brain barrier and optimum time of chemotherapy. Int J Radiat Oncol Biol Phys.

